# Supplementary material for: The interactome of intact mitochondria by cross-linking mass spectrometry provides evidence for coexisting respiratory supercomplexes
Source: Mol Cell Proteomics. 2017 Dec 8;17(2):216–32. doi: 10.1074/mcp.RA117.000470 (PMC5795388; doi:10.1074/mcp.RA117.000470)
Supplement: Supplemental Data [file supp_RA117.000470_133922_0_supp_23978_fzffwf.zip › spectra_annotation/mito_DR_spectra_annotation/39-1-6-1-11-1.pdf]

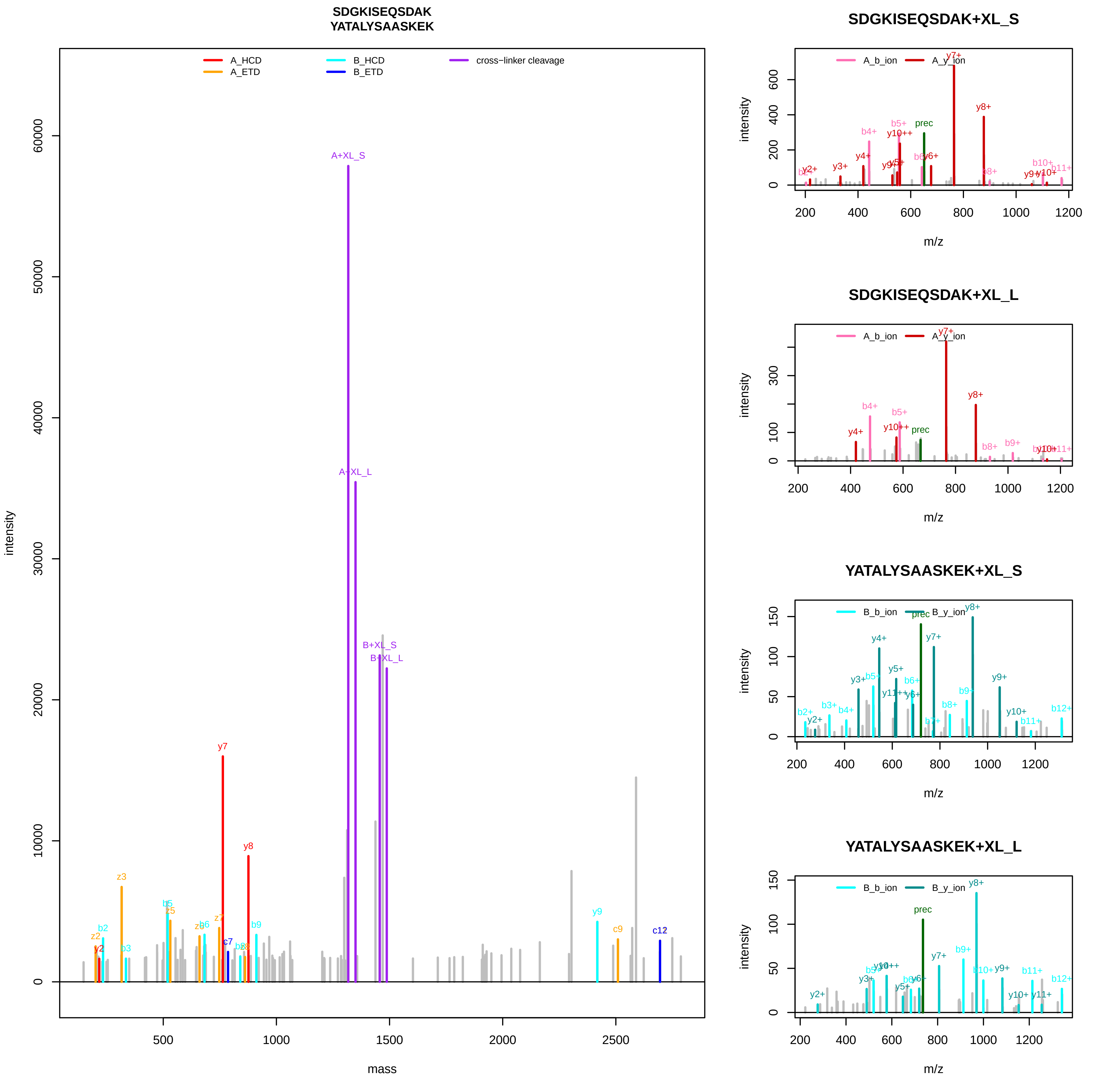
SDGKISEQSDAK
YATALYSAASKEK
A_HCD
A_ETD
B_HCD
B_ETD
cross−linker cleavage
A+XL_S
A+XL_L
B+XL_S
B+XL_L
intensity
mass
0
10000
20000
30000
40000
50000
60000
500
1000
1500
2000
2500
y7
y8
z3
b5
z5
z2
b2
y2
b3
z6
b6
z7
c7
b9
y9
c9
c12
SDGKISEQSDAK+XL_S
SDGKISEQSDAK+XL_L
YATALYSAASKEK+XL_S
YATALYSAASKEK+XL_L
A_b_ion
A_y_ion
B_b_ion
B_y_ion
prec
m/z
intensity
y7+
y8+
b4+
b5+
y10++
y4+
y3+
y2+
y6+
b8+
b9+
b10+
b11+
y9+
y10+
y11+
y5+
b3+
b6+
b7+
b12+
y11++
